# How do patients with head and neck cancer and low skeletal muscle mass experience cisplatin-based chemoradiotherapy? A qualitative study

**DOI:** 10.1007/s00520-024-08950-0

**Published:** 2024-10-28

**Authors:** Anouk W. M. A. Schaeffers, Maartje A. van Beers, Lot A. Devriese, F. W. José Klomp, Chantal F. M. Westerink - van den Brink, Ernst J. Smid, Remco de Bree, Caroline M. Speksnijder

**Affiliations:** 1 Department of Head and Neck Surgical Oncology, University Medical Center Utrecht, Utrecht University, G05.122, P.O. Box 85.500, 3508 GA Utrecht, The Netherlands; 2 Department of Medical Oncology, University Medical Center Utrecht, Utrecht University, Utrecht, The Netherlands; 3 Department of Radiotherapy, University Medical Center Utrecht, Utrecht University, Utrecht, The Netherlands; 4 Department of Oral and Maxillofacial Surgery and Special Dental Care, University Medical Center Utrecht, Utrecht University, Utrecht, The Netherlands

**Keywords:** Chemoradiation, Cisplatin, Skeletal muscle mass, Dose limiting toxicity, Head and neck cancer, Qualitative research

## Abstract

**Background:**

Patients with head and neck squamous cell carcinoma (HNSCC) face several physical, emotional, and psychological challenges throughout treatment. Cisplatin-based chemoradiotherapy (CRT) is an effective but toxic treatment, with an increased risk for toxicities in patients with low skeletal muscle mass (SMM). Consequently, these patients are anticipated to experience greater treatment-related difficulties. We aimed to explore the experiences of patients with HNSCC and low SMM regarding cisplatin-based CRT.

**Methods:**

A descriptive qualitative study was conducted, interviewing seven patients 3 months after CRT using a topic guide. Thematic analysis of semi-structured interviews was conducted, to create a multi-dimensional understanding of patients’ experiences during and after cisplatin-based CRT.

**Results:**

Prior to CRT themes included pre-treatment information, expectations towards treatment and trial, psychosocial circumstances, and supporting network. During CRT themes included toxicities, psychosocial impact, and supporting network. After CRT themes included reflection on period during CRT, psychosocial circumstances, informal support from networks and healthcare workers, and ongoing toxicities.

**Conclusion:**

Most patients experience cisplatin-based CRT as a life-changing and distressing life event but cope through various strategies and supporting networks. Tailored counseling, ideally with on-demand consultations, is recommended. No differences were noted in patients’ perceptions of their cisplatin regimen.

## Introduction

Head and neck squamous cell carcinoma (HNSCC) and its treatment can affect speech, swallowing, chewing, appearance, voice, hearing, social circumstances, and overall health, leading to a decreased quality of life (QoL) [1–5]. Multiple studies have shown the substantial effect of HNSCC and its treatment on QoL for both patients and caregivers [2–4, 6–9]. Patient-centered care encompasses shared decision-making, enhanced communication between healthcare providers and patients, and engaging informal caregivers such as family into the diagnostic and treatment process [10]. It has been proven that patient-centered care positively influences outcomes, such as survival and rehabilitation [11, 12]. Understanding patients’ preferences is essential, but may not be fully captured by pre-defined questionnaires [13].

Patients with HNSCC receiving radiotherapy (RT) and their caregivers face several physical and psychological challenges [14]. However, there is limited knowledge regarding the expectations and experiences of patients with HNSCC receiving primary cisplatin-based chemoradiotherapy (CRT), despite its significant impact on QoL [4]. Cisplatin-based CRT is a standard treatment for locally advanced HNSCC with curative intent. Despite its effectiveness, it is highly toxic, frequently leading to cisplatin dose limiting toxicities (CDLT) and also late toxicities, such as dysphagia [15, 16]. Patients with low skeletal muscle mass (SMM) are especially prone to these toxicities during and after CRT [17–20]. This group also faces worse survival rates, prolonged feeding tube dependency, and a higher risk of frailty compared to patients without low SMM [21–26]. Thus, it is important to explore the expectations and experiences of patients with HNSCC with low SMM regarding cisplatin-based CRT. Identifying these expectations and experiences could help healthcare workers in approaching this specific patient category. Understanding patient expectations is crucial to improve patient satisfaction and outcomes [27–29]. This qualitative study aimed to explore the expectations and experiences of patients with HNSCC and low SMM, regarding cisplatin-based CRT and the triweekly (100 mg/m^2^) or weekly (40 mg/m^2^) cisplatin regimen.

## Methods

This qualitative study was conducted according to the Standards for Reporting Qualitative Research (SRQR) [30]. The patients approached for the semi-structured interviews are part of the CISLOW study [31], which is a multicenter randomized clinical trial, that is approved by the Medical Ethics Committee (METC), registered in the Netherlands Trial Register (NL76533.041.21 and NL9217), and funded by The Netherlands Organisation for Health Research and Development (ZonMw; 10,140,021,910,002) [31].

### CISLOW study protocol

In the CISLOW study, patients with HNSCC and low SMM who are scheduled for cisplatin CRT are randomized to receive either a triweekly (100 mg/m^2^) or a weekly (40 mg/m^2^) cisplatin regimen. The primary outcome was the difference in CDLT between these regimens. SMM was assessed using routine baseline CT or MRI scans of the head and neck prior to the start of treatment [32–36]. The cross-sectional muscle area (CSMA) of the sternocleidomastoid and paravertebral muscles at the third cervical level was delineated using sliceOmatic software v5.0 (TomoVision, Canada). The corresponding CSMA at the third lumbar vertebra (CSMA_L3_) and lumbar skeletal muscle index (LSMI) were both calculated, using the following formulas [32]:

$$CSMA\; L3 \left({cm}^{2}\right)=27.304+1.363\times CSMA\; at\; C3 \left({cm}^{2}\right)-$$

$$0.671\times age\, (years)+0.640\times weight\, (kg)+26.442\times sex\, (sex=1\; for\; female,\; 2\; for\; male)$$

$$LSMI ({cm}^{2}/{m}^{2})=\frac{CSMA\; at\; L3\, \left({cm}^{2}\right)}{{Height\; (m)}^{2}}$$

Low SMM was defined as a LSMI < 43.2 cm^2^/m^2^ [18–20].

### Patient recruitment

Patients were recruited in the CISLOW study [31]. Inclusion criteria included (1) treatment with primary cisplatin-based CRT, (2) 18 years and older, and (3) fluency in Dutch. Exclusion criteria included (1) inability to give informed consent and/or mental disability, (2) ineligibility for triweekly cisplatin, (3) cisplatin as induction treatment or not in a primary setting, (4) diagnostic scans older than 2 months at the start of CRT, and (5) inability to measure SMM due to bilateral lymph node metastasis or artifacts. Only patients with low SMM, treated at the University Medical Center Utrecht (UMCU) who signed informed consent, were asked to participate in the interviews. Patients were interviewed 3 months after CRT until data saturation was reached, defined as no new information emerging in two consecutive interviews [37, 38].

### Interviews

Semi-structured telephone interviews were conducted by AS, a trained medical doctor. Interviews followed pre-defined topic guides, which were refined by AS, a head and neck medical oncologist (LD), a head and neck surgeon (RdB), and an epidemiologist (CMS) with experience in qualitative research. Interview topics covered [39] (1) information and guidance about the treatment, (2) support network, (3) experiences during treatment, (4) experiences after treatment, and (5) differences in daily life before and after the treatment. The last three categories were then subdivided into four categories, namely (1) physical, (2) cognitions, (3) social, and (4) emotional experiences [40].

At first, during the interviews, the participants were informed about why they were conducted and the logistics on data retrievement. Then, the patients were asked about their experiences before, during, and after the treatment and the patients’ supporting network, not necessarily in this order. Questions were asked open, to ensure the patients would not be influenced into a certain direction. Examples were “How did you experience the chemotherapy?” and “What were your expectations of the chemotherapy before the start of treatment?” When patients primarily provided answers about their physical feelings, the interviewer asked additional questions to explore their emotional or psychological experiences. Examples were “How did that make you feel emotionally?” and “How was your state of mind during therapy?” Moreover, detailed questions about adverse events were asked, such as “Have you experienced adverse events, and if so which, and how did this affect you?” When the patient had difficulties remembering adverse events, options were given based on current knowledge of common adverse events.

### Data analysis

Interviews lasted 45 to 75 min, were recorded, and were transcribed verbatim. The transcriptions of the interviews were shared anonymously with CMS after each interview, and AS and CMS amended the topic guide when necessary. Transcripts were uploaded anonymously into NVivo 14 [41]. Two researchers (AS and MvB, graduated as medical doctor, working as a PhD student at the Head and Neck Surgical Oncology Department, and trained for qualitative research conducting) independently analyzed data using a thematic analysis approach, supervised by CMS [42]. Thematic analysis was used to create a multi-dimensional understanding of the experiences of patients during and after cisplatin-based CRT [43]. The transcriptions were read thoroughly in order to find quotes, create codes, and corresponding themes. Themes were created based on time period, e.g., prior to CRT, during CRT, and after CRT. Codes, themes, and categories were discussed among AS, MvB, and CMS. The results were reviewed by a former patient with HNSCC who received CRT prior to the CISLOW study, to verify outcomes and reach consensus.

## Results

### Patient characteristics

Data saturation was reached after the conduction of seven interviews. Patient and treatment characteristics are summarized in Table 1. The average age was 62 ± 6 years; three out of seven patients were female. Three patients received weekly cisplatin, and four patients received triweekly cisplatin. CDLT occurred in two patients receiving the triweekly regimen and in one patient on the weekly regimen. The average cumulative cisplatin dose was 257 ± 44 mg/m^2^ cisplatin. CDLT had different causes, e.g., ototoxicity, anorexia, and neutropenia, but no nephrotoxicity was observed. Adverse events that did not lead to CDLT were more common, ranging from Common Terminology Criteria for Adverse Events (CTCAE) grade 1 (three events) to CTCAE grade 3 (six events), as depicted in Table 2 [39]. No patients experienced disease recurrence between the end of CRT and the interviews, which were conducted 3 months post-treatment.

**Table 1:** Patient and treatment characteristics

Patient	Gender	Disease stage according to UICC	Tumor location	Cisplatin treatment scheme	Number of received cisplatin cycles	Cumulative cisplatin dose in mg/m^2^	DLT	DLT cause
1	Male	Stage I	Oropharynx	Weekly 40 mg/m^2^	7	280	No	
2	Female	Stage III	Oropharynx	Triweekly 100 mg/m^2^	3	300	No	
3	Male	Stage I	Oropharynx	Triweekly 100 mg/m^2^	2	200	Yes	Ototoxicity
4	Male	Stage I	Oropharynx	Weekly 40 mg/m^2^	7	280	No	
5	Female	Stage I	Oropharynx	Weekly 40 mg/m^2^	6	240	Yes	Ototoxicity and anorexia
6	Male	Stage III	Oropharynx	Triweekly 100 mg/m^2^	3	300	No	
7	Female	Stage IVa	Unknown primary	Triweekly 100 mg/m^2^	2	200	Yes	Neutropenia

UICC: Stage is according to the Union for International Cancer Control criteria

*DLT* dose limiting toxicity

**Table 2:** Adverse events according to Common Terminology Criteria for Adverse Events version 5.0

Adverse events	Amount of patients suffering event	Grade according to CTCAE
**Acute kidney injury**	2	3
**Anorexia**	1	3
**Neutropenia**	1	3
**Hearing impaired**	2	2
**Infection**	1	1
**Mucositis**	2	2 and 3
**Nausea**	2	3
**Thromboemboli**c** event**	1	2
**Tinnitus**	2	1

*CTCAE* Common Terminology Criteria for Adverse Events version 5.0, *DLT* dose limiting toxicity

### Overview of results

**Fig. 1:**
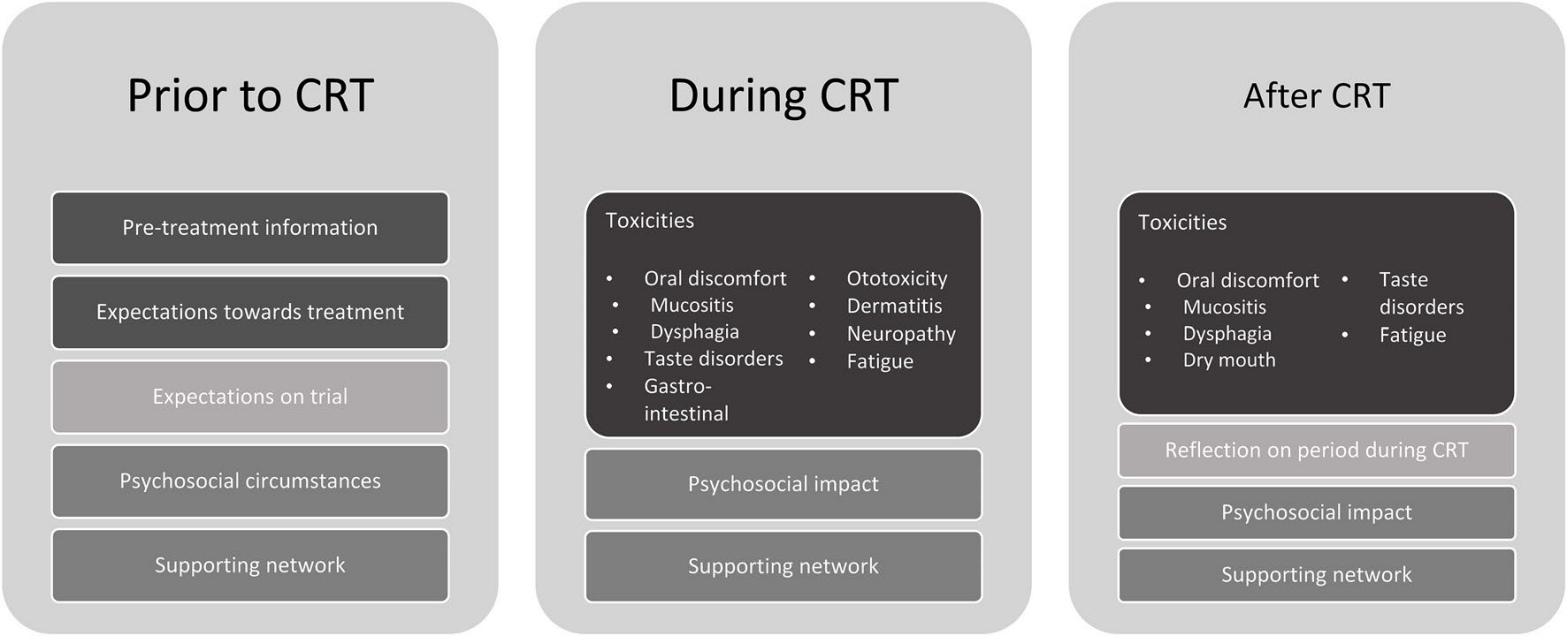
Identified themes and subthemes in data analysis. CRT, cisplatin-based chemoradiotherapy

The timing of CRT (before, during, or after) was a key factor in the themes identified. Prior to CRT, themes were pre-treatment information, treatment and trial expectations, psychosocial circumstances, and supporting networks. During CRT themes were toxicities, psychosocial impact, and supporting network. After CRT themes were reflections on the CRT period, psychosocial circumstances, both informal supporting networks and support from healthcare workers, and ongoing toxicities. The identified themes and subthemes, related to patients’ expectations and experiences around CRT are depicted in Fig. 1.

Corresponding quotes, translated from Dutch to English by AS, to the identified themes are presented in Table 3, with specific quotes on toxicities during and after treatment in Tables 4 and 5.

**Table 3:** Quotes illustrating patients’ experiences during chemotherapy

Themes	Subthemes	Patient	Quote number	Quote
**Prior to CRT**	Pre-treatment information	4	1	I thought the hospital was very well organised from the start. I was surprised at how well everything was coordinated, how quickly appointments were made, and how much information you were given
		6	2	Let me think, actually, that your taste deteriorated so badly and my smell disappeared. Those were things I hadn’t been told beforehand, and I would have like to know that
	Expectations towards treatment	2	3	I have surrendered and if the doctors say it is treatable and then we go for it. That’s the only thing I owe myself and the people around me
		4	4	I was especially afraid of nausea symptoms, because I’ve seen that people with that
	Expectations on trial	1	5	I was happy to be included in the study… It’s good that research is being done to improve this kind of treatment. Seven small doses of chemo seem less toxic to the body than three large doses in my opinion. With the triweekly schedule you have to stay 1–2 nights, so the fact weekly cisplatin could be given in a day treatment seemed to me as a big advantage
	Psychosocial circumstances	3	6	When the ear, nose and throat specialist told me that he had bad news and that my wife had to be there, I thought that moment was the worst of everything. I thought I had to stop my hobbies and sell my motorbike. The doctor ensured me I should not act too quick on this
	Supporting network	4	7	Fortunately, my wife is a bit familiar with cancer research. She took lots of notes and worked it all out. Thanks to that, I was able to process the information
**During CRT**	Toxicities			*See *Table 4
	Psychosocial impact	1	8	I didn’t really function socially, I just sat at home and didn’t need visitors. Life stopped for a while, I was at home and not working. How does that affect your life? The treatment, but not only, also the physical aspects plus the mental, the whole thing. It affected my life enormously. You don’t have a life anymore
		6	9	After I got the second cisplatin gift and covid, my social life actually dropped to zero right away, we were just the two of us and I didn’t want to see anyone anymore either
	Supporting network	2	10	Our bond was already strong, but it has become even stronger between my son and me
		7	11	My husband really likes cooking, so he took very good care of me. And if I didn’t eat, he would come and say: you have to eat again
**After CRT**	Toxicities			*See *Table 5
	Reflection on period during CRT	1	12	I can’t compare, of course. I have not undergone this standard of 3 chemos so it is hard to compare. There is also a disadvantage. You have 7 chemos with 7 infusions and otherwise you only have that three times. With the infusion, the needle placement didn’t work in one go, so I had to undergo several attempts. Didn’t go so smoothly…
		4	13	The weekly regimen, I did not experience that intensity as a negative thing. That once a day radiotherapy, and once a week chemotherapy brings structure to your life
	Psychosocial impact	5	14	Anyway, I feel gratitude that I was helped. It was a bit tough, but then the time went really fast because you’re on the road every day and the time goes fast anyway, so afterwards I thought it wasn’t so bad looking at how fast it went
		3	15	It did affect me a lot, but not in a negative way. I don’t know if you’ve ever had a car breakdown, you’d rather not experience that. But when you experience it a lot of things often happen: we had a rented car, we slept at people’s houses because they felt sorry for us and in short a lot more happens than when everything runs smoothly. And that happened to me again now, I would have preferred not to have it at all but now it has enriched my whole being
	Supporting network	6	16	In fact, I’ve been having a lot of scary dreams about the mask I’ve been wearing, and it’s coming back to me in my dreams. She *[the therapist]* wants to help me process it
		3	17	I was also supported by the specialized nurse, with whom I could call if I wanted to

**Table 4:** Quotes illustrating patients’ experiences of toxicities during chemoradiotherapy

Toxicities during CRT	Subcategories	Patient	Quote number	Quote
**Oral discomfort**		3	17	I had up to thirty bins full of papers with slime in my mouth, that white foamy slime. I almost forgot about it! It was terrible
	Mucositis	1	18	My gums, my palate and the lining of my mouth became inflamed. Not to the worst degree, according to the radiotherapist. But it was definitely disabling. I could still eat, but eating and drinking was painful and swallowing took a long time
	Dysphagia	2	19	Swallowing was very painful because the inner lining of my throat was destroyed by the radiotherapy. The swallowing was actually the most painful thing
**Taste disorders**		3	20	I could no longer put food in my mouth. I noticed that in the 4th week of radiotherapy…
**Gastro-intestinal**		1	21	What I had quite quickly and went through during the two months of treatment was stomach pain. A lot of stomach pain
	Emesis and diarrhea	1	22	Because of the medication against the nausea, I had a lot of constipation. I had to swallow things for that, magnesium and sachets of powder, and at some point I couldn’t take those either, nasty stuff
**Ototoxicity**	Hearing	6	23	My hearing got very bad. I had a test before and after the treatment and it showed that my hearing had deteriorated a lot
	Tinnitus	3	24	Look, if you can’t hear anything anymore, it’s a disaster, or a beep that loud, it’s a madhouse. I won’t be able to cope if the beep would have been even louder
**Dermatitis**		1	25	The skin on my neck and throat was quite burned, like an extreme sunburn, peeling, it felt a bit like burning. It wasn’t too bad, the skin didn’t open up, it recovered quite quickly
**Neuropathy**		3	26	I still have neuropathy, in my feet and especially in the big toes, I have a numb feeling, tingling. I can still control it. But it’s okay

**Table 5:** Quotes illustrating patients’ experiences of toxicities after chemoradiotherapy

Toxicities after CRT	Subcategories	Patient	Quote number	Quote
**Oral discomfort**	Mucositis	1	27	I found the two weeks after the treatment very painful
	Dysphagia	7	28	Your physical complaints with your throat, and I still feel that I have given up a lot in terms of fitness
	Dry mouth	7	29	I have no improvement, in fact I wake up three times during the night because of my dry mouth and you feel like your tongue is sticking to your palate
**Taste disorders**		1	30	Sweet tastes as wry or sour, but okay, well. I found this very annoying, the most annoying side effect in fact, and I still suffer from it. It has driven me to mild despair
		2	31	The worst side effect is that I have no taste at all
		5	32	The most annoying side effect now is taste
		6	33	Regularly disappointing and sometimes a little sad. Damn, I miss it a lot
		7	34	The taste was really gone very quickly, completely lost. That was the worst thing. In the beginning I was very worried about it, when I couldn’t taste anything, I thought ‘oh dear, if it stays like that, what’s the quality of life going to be,’ because if you can’t taste or like anything, my wife and I love good food

### Prior to CRT

Before starting CRT, patients appreciated the coordinated, multi-disciplinary structure where they visited various specialists over a few days (e.g., otolaryngologist, head and neck oncologist, radiation oncologist, specialized nurse, dentist, and dietician). However, the amount of information given was sometimes overwhelming (quote nr. 1). Patients were unaware of the severity of some side effects, such as taste disorders (different taste, no taste, metal taste) (quote nr. 2). Patients were concerned about known side effects like nausea, but were willing to start treatment, driven by the hope to be cured (quote nr. 3 and 4). Patients had a positive view to the CISLOW study, citing reasons such as contributing to future research, the potential of fewer toxicities, and the gut feeling that seven lower doses might be less toxic (quote nr. 5). At the time of the diagnosis and treatment, patients felt motivated and more positive towards the start of treatment, because the doctor was realistic about treatment and survival (quote nr. 6). The presence of relatives during appointments was considered helpful, as they could take notes and assist in processing the information (quote nr. 7).

### During CRT

During CRT, most patients reduced their participation rate in social activities due to a lack of desire (quote nr. 8 and 9). However, relationships with relatives and household members strengthened, with patients valuing their support during this challenging period. It was helpful when relatives took care of the food preparations, ensuring the patients ate enough, which was appreciated (quote nr. 9,10, and 11).

Toxicities varied among patients, but fatigue was universally reported. Oral discomfort consisted of excessive mucus production (quote nr. 17); mucositis was significantly impacting QoL (quote 18) and dysphagia (quote 19). Food structures were experienced differently (quote nr. 20). One patient reported severe gastric pains (quote nr. 21). Nausea, emesis, diarrhea, and constipation were all common side effects (quote nr. 22) either caused by CRT or medication against side effects of CRT. Hearing loss and tinnitus were also reported, with one patient expressing relief that the tinnitus was not more severe (quote nr. 23 and 24). Dermatitis, reported by one patient, was manageable and healed quickly after the end of RT (quote nr. 25). Peripheral sensory neuropathy was present but acceptable (quote nr. 26). No discernible differences in the type, number, or severity of subjective side effects or experiences between the weekly cisplatin and the triweekly comparison group were observed.

### After CRT

Reflecting on CRT, patients reported that the weekly regimen brought structure in their lives (quote nr. 13). In one patient, the placement of the intravenous needle was challenging (quote nr. 12). Patients expressed gratitude for receiving treatment. They noted that the CRT period passed quickly but was sometimes overwhelming (quote nr. 14). Experiencing sickness and receiving CRT also gave new life experiences neither wholly positive nor negative (quote nr. 15). Some reported nightmares about the RT mask and fear for recurrence, but support from their network helped them cope with the mask (quote nr. 16). One patient found it particularly helpful to talk with the specialized nurse (quote nr. 17).

After CRT, some symptoms persisted, which patients had not expected. Mucositis worsened post-treatment (quote nr. 27), dysphagia continued, overall fitness remained low, and fatigue persisted (quote nr. 28). Patients hoped for symptom improvement, but issues like dry mouth lingered, disrupting sleep (quote 29). The most distressing and lingering side effects were taste disorders, which significantly impacted patients’ QoL (quote nr. 30–34).

## Discussion

In this qualitative study, we explored the experiences of patients with HNSCC and low SMM, regarding cisplatin-based CRT. Patients described this HNSCC treatment as a life-changing and distressing event. Per time period (e.g., before, during, and after CRT), several themes emerged, addressing these experiences and perceptions. Patients did not perceive differences in cisplatin regimens, noting they could not compare treatment regimens since they had only received one.

Pre-treatment information was perceived as helpful and complete, aligning with existing literature [44]. Some patients found it overwhelming, highlighting the variation in information preferences among patients. For example, some patients with HNSCC prefer qualitative prognostic information, while others seek precise survival statistics [45]. Patients were surprised about persistent side effects and expressed a desire to have known about this earlier, as noted in other research [46]. This suggests a need for tailored approaches to information delivery and early psychological support, including honest discussions about coping, HNSCC, and its treatment [45, 47]. A specialized nurse could provide this support through an outpatient consultation with newly diagnosed patients. During this session, the nurse could discuss expected nausea and potential long-term effects (e.g., dry mouth, taste alterations, and dysphagia) and inform patients that some side effects, such as mucositis, might temporarily worsen post-treatment.

In a study on expectations across several cancer types, patients had optimistic expectations on the treatment outcomes; however, females particularly expected hair loss and taste alterations. The patients also anticipated nausea, fatigue, and weight loss [48]. This was similar to our findings where all patients expected a challenging treatment with nausea and fatigue. Some patients reported their expectations were accurate, while others reported under- and overestimations of side effects. It is likely that patients adjusted their expectations and experiences during treatment to cope with outcomes, leading to different expectations post-therapy compared to pre-therapy [49].

The standard cisplatin regimen in CRT is triweekly 100 mg/m^2^ per body surface area (BSA), but weekly 40 mg/m^2^ per BSA is also common [50–61]. CDLT frequently occurs, reducing cumulative doses, which are crucial for improved survival and locoregional control [50–53]. Patients with HNSCC and low SMM are more prone to CDLT than those with normal SMM [20, 62]. It is believed that a weekly regimen of 40 mg/m^2^ per BSA could reduce CDLT and allow for a higher cumulative cisplatin dose. In the CISLOW study, from which we included the patients in these interviews, patients with HNSCC and low SMM were randomized between weekly and triweekly cisplatin regimens [31]. We observed no differences in patient experiences between these regimens, consistent with another study stating comparable QoL [63]. However, the small sample size (*n* = 3 and *n* = 4) may have limited our ability to detect differences. One drawback of the weekly regimen was the more frequent placement of intravenous needles which caused difficulties for one patient.

Emotional and social consequences of the disease, treatment, and toxicities were commonly reported, with one patient describing it as “no longer having a life.” In our study, patients reported reduced eating abilities, leading to social isolation and emotional distress, consistent with research on the emotional impact of eating disabilities in various cancer types [64].

Patients with HNSCC may be more prone to an increased emotional burden and psychological distress before and after treatment, especially patients with weaker social support systems [65]. A systematic review found a 63% prevalence of depression among patients with HNSCC undergoing RT, with distress increasing during treatment, especially in those with a low social support [66]. In our study, patients reported stopping working, reducing social activities, and experiencing social isolation. However, many noted that their bonds with loved ones strengthened over the period of diagnosis, during and after treatment, which is in line with other studies [47, 67]. Unfortunately, also conflicts can arise. Communication skills training is recommended to help couples face the diagnosis and treatment together [14]. It is important to address the needs of the patients and caregivers, who are both affected by the disease and treatment. Over 70% of informal caregivers have at least one unmet care need during the period of therapy, and there was an association between unmet needs in patients and their caregivers [68]. Caregivers often neglect their own well-being to prioritize the patient [69], highlighting the need of a multi-dimensional approach to ensure both patient and caregiver receive adequate support.

Coping strategies mentioned in the interviews mirrored those in other research [47].

Patients expressed worries during diagnosis but also showed determination to fight. One patient focused on finding positivity amid the challenges, which has been postulated as an effective self-management [70]. A strong support network was essential, providing practical help, like transportation and emotional support. Access to a nurse specialist, available for consultation as needed, was highly valued by patients, aligning with recent guidelines that emphasize the role of nurse specialists in improving patient experiences during cancer treatment [71, 72].

A particular distressing aspect was the use of the RT mask, which caused long-term anxiety and nightmares for some patients. The mask is used to immobilize patients during RT, can elicit feelings of distress, and is described as the worse part of the treatment [73–75]. While anxiety about the mask reduces during treatment [73], the timing and intensity of this anxiety vary, making it challenging to identify a standard screening moment [76]. Effective strategies to reduce mask-related anxiety include clear communication, providing visual information, and allowing patients more control over music selection during treatment [73, 74, 76]. Additionally, fear of cancer recurrence is common, affecting over 50% of patients with HNSCC after treatment, and is often an unmet need in cancer follow-up, persisting months after treatment [77–79].

During treatment, patients experienced a subjective decline in physical functioning and several toxicities. Symptoms typically worsen over time, with early stage issues like xerostomia, decreased appetite, and taste disorders often giving way to later-stage problems such as sores and dysphagia [80]. Patients reported that post-treatment symptoms could be as invalidating as those experienced during treatment, aligning with previous research that found the highest symptom scores at treatment’s end, followed by a recovery period [81].

Dysphagia and xerostomia are well-known side effects with a severe burden to the patient and their QoL, which aligns with our study [82, 83]. Mucositis was also mentioned as a disabling side effect, which is in agreement with literature observing an 80% rate, causing severe mouth and throat pain which affected QoL [84].

Ototoxicity, manifesting as hearing loss and/or tinnitus, was also reported. High rates of tinnitus are reported in cancer survivors after neurotoxic chemotherapy such as cisplatin [85], and depression and anxiety are related to ototoxicity [86]. Patients stated that if they had experienced more ototoxicity, it would have been unbearable for them. This can be anticipated from other research that showed that ototoxicity affects daily life significantly [87]. Currently used criteria systems such as the CTCAE are less focused on the effects of ototoxicity on daily life and the effects of cisplatin on the ultra-high frequencies. Therefore, the incidence of ototoxicity affecting QoL in patients might be underestimated and undervalued [88–90]. Research on the subjective severity of hearing loss would be of interest [90].

Nausea and vomiting were also reported, which are well-known side effects of cisplatin. A study showed an incidence of nausea and vomiting of 48% in females and 26% in males treated with cisplatin over 50 mg/m^2^ on a triweekly base. They reported a 25% and 13% incidence in, respectively, females and males treated with cisplatin less than 50 mg/m^2^ on a weekly basis [91]. Patients were afraid of facing nausea, because they had known people who suffered from it during chemotherapy regimens, which is in agreement with the literature. Nausea and vomiting have a detrimental effect on QoL [92–95]. Fatigue is also described as a complication of RT and CRT in HNSCC with a detrimental effect on QoL. In our study, patients reported their fatigue as an influencing factor on their daily life [96, 97]. One study found an increase in fatigue at the end of CRT, followed by gradual improvement [97].

The most burdening side effects were taste disorders. Taste disorders are common and occur in 77% of patients with oral cancer receiving CRT [98] and in more than 80% of patients with oropharyngeal cancer [99]. The onset of these symptoms varied, but often developed in the first weeks of treatment, which has also been shown in previous research [80]. Symptoms often recover at 3 until 12 months after therapy, but unfortunately, some patients develop a chronic taste disorder which might be attributed to persistent damage to the chorda tympani or taste buds [100]. The severity and perception of the taste disorder varied which is in line with other research. One study showed that sweet flavors are perceived as tastier than spicy and bitter tastes [101], while others report sweet as the most altered taste [102, 103], which emphasizes the varying effects therapy can have on patients their taste abilities. Metal taste in the mouth is a familiar problem in chemotherapy; however, the mechanism behind it is not exactly understood [104]. Taste disorders are associated with malnutrition [105]. One patient reported improved taste and consequently intake when someone else prepared the food, which is in line with research that stated that the responsibility for cooking was associated with taste and smell disorders [106]. Taste disorders can affect QoL; hence, research towards the prevention and treatment of taste disorders is warranted [107, 108].

The strengths of this study are its relatively uniform patient population, in contrast to other qualitative studies which mostly included a range of patients with HNSCC treated with surgery, CRT, and/or RT in one [47, 49, 67, 70, 109], making the outcomes of this study more applicable to clinical practice for patients with HNSCC treated with CRT. However, the inclusion of only patients with low SMM may have possibly biased outcomes, as these patients with low SMM are frailer and may experience more side effects. A limitation to this study could be the sample size; thematic saturation suggests the study is sufficient to answer the research question, though further research with a larger cohort could help establish guidelines to improve patient and caregiver experiences.

In concurrent CRT, cisplatin acts as a radiosensitizer [56, 57, 110], potentially exacerbating RT-induced toxicities. Therefore, our conclusions are specific to concurrent cisplatin-based CRT and may not apply to cisplatin monotherapy, which is not a curative option for patients with HNSCC [61].

Cancer treatment often prioritizes survival, but there is no consensus on whether patients value survival over functionality [45, 109]. It likely depends on the patient’s current situation, including norms, values, and life standards [109, 111]. Pre-treatment individualized counseling is, in our opinion, essential to assess side effect severity and balance survival with QoL, using risk-estimating models for both weekly and triweekly cisplatin regimens. The models should calculate the risk of oto-, nephro-, and neurotoxicity; CDLT; oral discomfort; taste disorders; and an estimation of overall QoL.

Counseling should continue throughout treatment to address new challenges, with on-demand consultations providing necessary support. The post-treatment period, often as challenging as the treatment itself, is an underemphasized phase. More guidance through on-demand consultations, addressing everything from daily life problems to CRT-specific toxicities, would be beneficial. Support should extend beyond the patient to include their entire support system.

In conclusion, CRT is a life-altering event requiring structured care. Support from caregivers and nurse specialists can positively influence patients’ experience, and thorough follow-up with on-demand consultations to address post-CRT toxicities is recommended, especially for those with less supportive networks.

## Data Availability

No datasets were generated or analysed during the current study.
